# Endoscopic thyroid surgery via a breast approach: a single institution’s experiences

**DOI:** 10.1186/1471-2482-14-49

**Published:** 2014-08-05

**Authors:** Yong-Seok Kim, Kyu-Hwa Joo, Sun-Cheol Park, Kee-Hwan Kim, Chang-Hyuck Ahn, Jeong-Soo Kim

**Affiliations:** 1 Department of Surgery, Uijeongbu St. Mary’s Hospital, College of Medicine, The Catholic University of Korea, Uijeongbu, Korea

**Keywords:** Endoscopic thyroidectomy, Thyroidectomy, Thyroid carcinoma

## Abstract

**Background:**

Thyroid carcinoma in young women is rapidly increasing, and cosmesis plays an important role in thyroid operations. Various endoscopic thyroid surgery approaches have been performed, and their application has recently been extended. We performed endoscopic thyroid surgeries via a breast approach since 1999. Herein, we evaluate the safety of this approach and identify the outcomes for differentiated thyroid carcinoma.

**Methods:**

A total of 452 consecutive patients with thyroid and parathyroid disease underwent endoscopic thyroidectomy via a breast approach at Uijeongbu St. Mary’s Hospital between November 1999 and December 2012. The inclusion criteria for endoscopic thyroidectomy included a benign tumour less than 4 cm in diameter, malignant thyroid nodules less than 2 cm, and no evidence of lymph node metastasis or local invasion. We analysed the clinicopathologic data and surgical factors of this approach.

**Results:**

The mean age of the patients was 38.4 ± 10.6 years (range 11-73 years). The mean tumour size was 2.12 ± 1.17 cm (range 0.1-4 cm). The final tumour pathologies included papillary carcinoma (n = 120), follicular carcinoma (n = 8), nodular hyperplasia (n = 266), follicular adenoma (n = 43), and Hüthle cell adenoma (n = 4). The mean postoperative hospital stay was 3.8 ± 1.3 days (range 1-17 days). Temporary and permanent hypoparathyroidism requiring calcium and vitamin D supplementation developed in 32 (7.1%) and 4 (0.9%) patients, respectively. Transient vocal cord paresis occurred in 20 (4.4%) patients.

**Conclusions:**

For patients with benign and low-risk malignant thyroid disease, endoscopic thyroidectomy via a breast approach is a safe, feasible, and minimally invasive surgical method with minimal complications.

## Background

Conventional open thyroidectomy remains the treatment of choice for benign and malignant thyroid nodules, but the surgery requires a long incision line on the neck and leaves a long scar on the lower anterior neck. This incision may lead to prominent scarring that can develop into keloid or hypertrophic scars and lead to paraesthesia or hypaesthesia [1].

With the development of laparoscopic and endoscopic surgery, thyroid and parathyroid surgery has recently been attempted using an endoscopic approach. Since endoscopic parathyroidectomy and thyroidectomy were first introduced by Gagner and Huscher et al. [2,3], various endoscopic thyroid surgery approaches have been devised, including cervical, axillary, breast, and anterior chest approaches.

Endoscopic thyroidectomy was initially performed in patients with benign thyroid nodules. As surgical experience was accumulated, the application of endoscopic thyroidectomy was extended to patients with early cases of thyroid cancer.

We have previously reported on the feasibility of endoscopic thyroidectomy compared with conventional open thyroidectomy [4]. Since 1999, we have operated on 452 patients using endoscopic thyroidectomy via a breast approach. The results of our 13-year experience are presented in this article. The purpose of this study was to evaluate the safety and surgical outcomes of endoscopic thyroidectomy and to analyse the clinicopathologic features, types of operation, operation time, and complications of this surgical approach.

## Methods

### Patients

A total of 452 consecutive patients with thyroid and parathyroid disease underwent endoscopic thyroidectomy via a breast approach at Uijeongbu St. Mary’s Hospital between November 1999 and December 2012. All operations were performed by a single surgeon (JSK). Written informed consent was obtained from the patients and their families. This study was reviewed and approved by the institutional review board at Uijeongbu St. Mary’s Hospital (UC13RISI0156).

Preoperative diagnoses of thyroid nodules were made by ultrasonography and ultrasonography-guided fine needle aspiration cytology (FNAC). If FNAC identified malignancy or atypical cells that were suspicious for malignancy, computed tomography was performed to identify the tumour location, invasion, and central lymph node metastasis.

The indications for endoscopic thyroidectomy were as follows: 1) benign tumour with the largest diameter less than 4 cm in size (as estimated by preoperative ultrasonography); 2) malignant thyroid nodules less than 2 cm in size; and 3) no evidence of lymph node metastasis or local invasion. Patients who had undergone neck surgery or irradiation were excluded. Additional exclusion criteria were lateral lymph node metastases, extrathyroid extension, invasion into adjacent organs, or suspicion of distant metastasis on the preoperative imaging.

We performed prophylactic ipsilateral central lymph node dissection for differentiated thyroid carcinoma. All patients with differentiated thyroid carcinoma were treated with levothyroxine to suppress thyroid-stimulating hormone, and all patients were regularly followed by ultrasonography and thyroglobulin at 6-month intervals. Differentiated thyroid carcinomas were described using tumour-node-metastasis (TNM) staging on the basis of the 7^th^ edition of the recommendations of the American Joint Committee on Cancer (AJCC).

### Surgical technique

**Figure 1:**
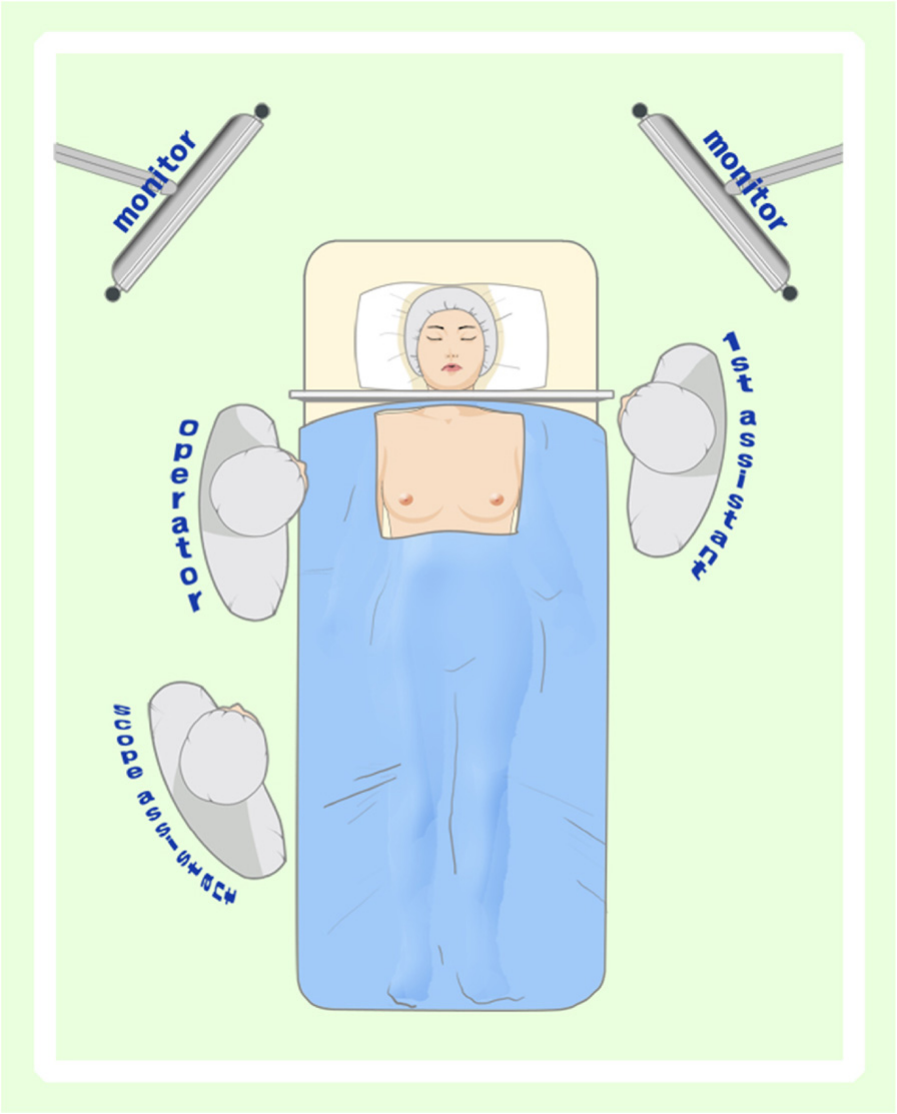
Operator and assistant positions during endoscopic thyroidectomy via a breast approach.

**Figure 2:**
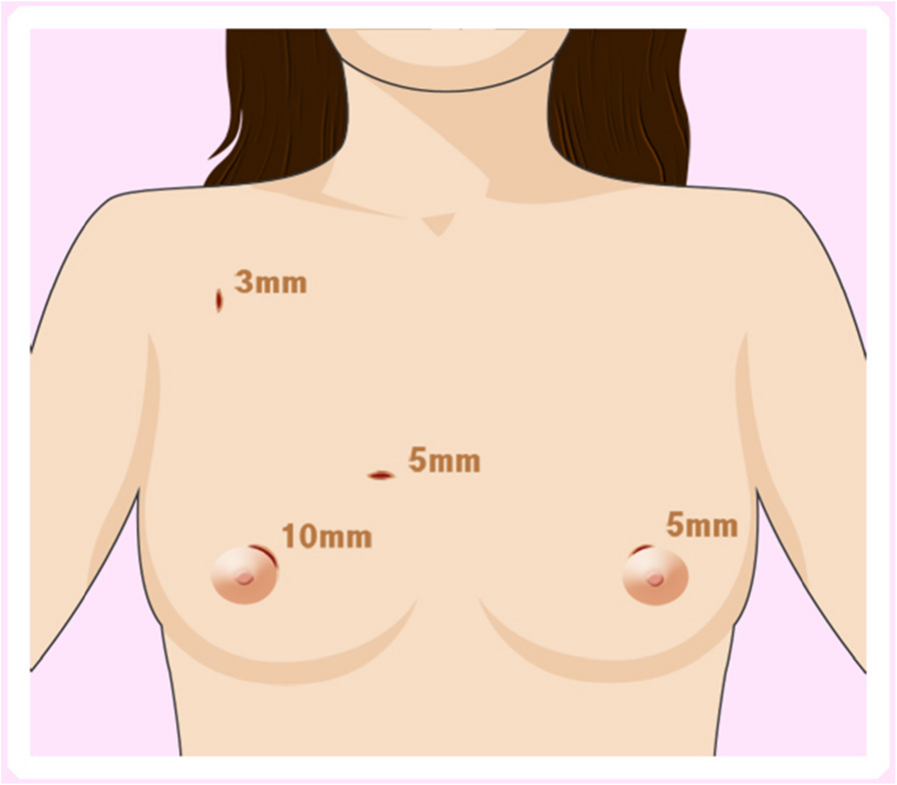
Illustration of the trocar site in endoscopic thyroidectomy via a breast approach.

**Figure 3:**
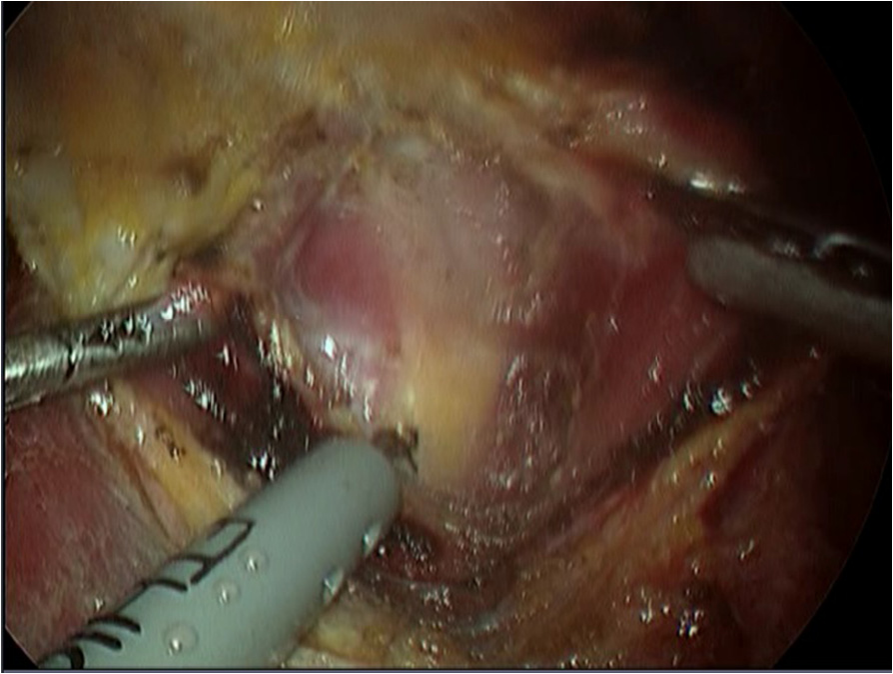
Division of the strap muscle.

**Figure 4:**
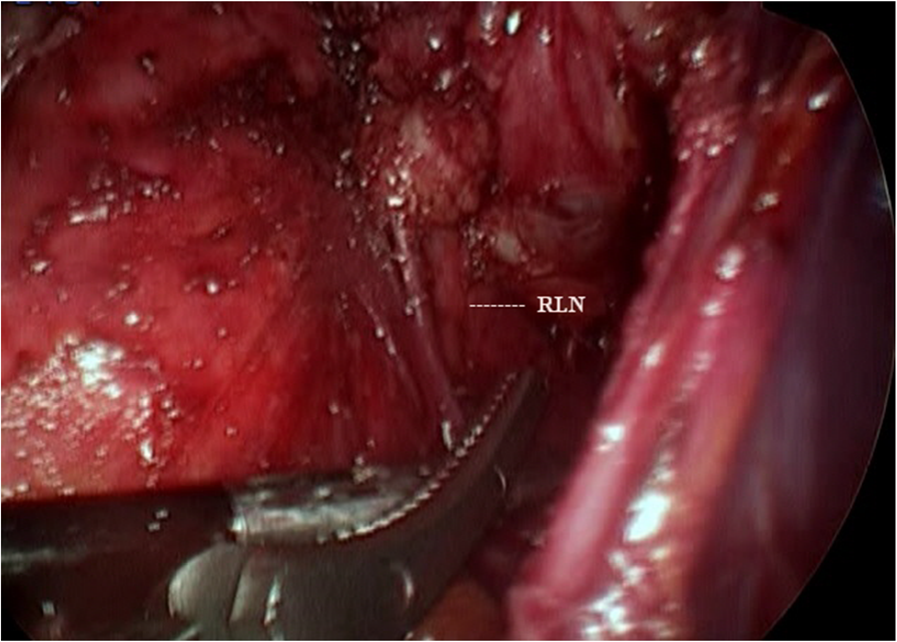
Identifying the recurrent laryngeal nerve.

All patients were prepared for endoscopic thyroidectomy under general anaesthesia. After the patient was placed in a supine position, a pillow was placed beneath the shoulder to extend the head and neck. The operator and scope assistant stood on the right side of the patient, the first assistant stood on the left side of the patient, and the monitors were placed on both sides of the patient (Figure 1). To facilitate dissection and reduce bleeding, approximately 50 ml of saline solution (including 1 ml epinephrine and 20 ml bupivacaine) was injected into the subcutaneous layer of the anterior chest and the subplatysmal space in the neck. A 10-mm incision was made on the upper edge of the areola on the right side, a 5-mm incision was made on the upper edge of the areola on the left side, a 5-mm incision was made in the right parasternal region, and a 3-mm incision was made in the right axillary area (Figure 2). Initially, the flaps were dissected bluntly with a glass rod bar. After the blunt dissection, ports were inserted through each incision. Carbon dioxide gas was injected with a pressure of 6 mmHg. A 30-degree 5-mm rigid endoscope (Olympus, Tokyo, Japan) was inserted through the trocar in the right parasternal region. To create adequate working space, an additional dissection was performed with endoscopic shears and hooks (ENDOPATH Probe Plus II, Ethicon Endo-Surgery, Inc., Guaynabo, Puerto Rico, USA) by electrical cauterization. The working space was extended from the anterior chest to the thyroid cartilage level and laterally to the medial edge of each of the sternocleidomastoid muscles. After the extension of the flap, a midline division was made between the strap muscle from the thyroid cartilage to the sternal notch (Figure 3). Ultrasonic shears (Harmonic ACE, Ethicon Endo-Surgery, Inc., Guaynabo, Puerto Rico, USA) were then used to expose and dissect the thyroid gland from the inferior pole. The inferior thyroid vessel and the isthmus of the thyroid gland were divided using ultrasonic shears. After a careful dissection of the inferolateral aspects of the thyroid gland, the recurrent laryngeal nerve and the inferior parathyroid gland were identified (Figure 4), and the inferior thyroidal artery and the middle thyroidal vein were identified and divided. The superior pole of the gland was dissected, and the superior thyroid vessel was divided with ultrasonic shears. After the recurrent laryngeal nerve was identified, the thyroid gland was separated from the trachea and Berry’s ligament was carefully divided. After being placed in an endo-bag (Sejong Medical, Seoul, Korea), the specimen was pulled out through the endoscope port site. A frozen section of the specimen was examined intraoperatively for pathologic confirmation. The cavity was cleaned with a saline solution before a meticulous haemostasis was performed. The strap muscles were approximated with an absorbable suture. A 100-cc Jackson-Pratt drainage tube was left in the operative bed through the endoscope port site.

## Results

The clinicopathologic characteristics of the 452 patients who underwent endoscopic thyroidectomy are presented in Table 1. The mean age of the patients was 38.4 ± 10.6 years (range 11-73 years). The mean tumour size was 2.12 ± 1.17 cm (range 0.1-4 cm). Four hundred thirty-seven (96.7%) patients were female, and 15 (3.3%) patients were male. The final pathologies of the tumours included papillary carcinoma (n = 120), follicular carcinoma (n = 8), nodular hyperplasia (n = 266), follicular adenoma (n = 43), Hüthle cell adenoma (n = 4), parathyroid adenoma (n = 3), Hashimoto’s thyroiditis (n = 1), diffuse hyperplasia (n = 4), granulomatous thyroiditis (n = 2), and hyaline fibrosis (n = 1). The mean postoperative hospital stay was 3.8 ± 1.3 days (range 1-17 days).

**Table 1:** Clinicopathologic characteristics of 452 patients who underwent endoscopic thyroidectomy via a breast approach

**Characteristics**		**Results (n = 452)**
Age (years)		38.4 ± 10.6 (range 11-73)
Gender	Female	437 (96.7%)
	Male	15 (3.3%)
Size (cm)		2.12 ± 1.17 (range 0.1-4)
Pathology	Papillary carcinoma	120 (26.5%)
	Follicular carcinoma	8 (1.8%)
	Nodular hyperplasia	266 (58.8%)
	Follicular adenoma	43 (9.5%)
	Hüthle cell adenoma	4 (0.2%)
	Parathyroid adenoma	3 (0.9%)
	Others	8 (1.8%)
Mean postoperative hospital stay (days)		3.8 ± 1.3 (range 1-17)

The mean operation times by operation type were as follows: total thyroidectomy (n = 3), 156.6 ± 48.0 min; total thyroidectomy with central lymph node dissection (n = 47), 171.6 ± 38.9 min; near-total thyroidectomy (n = 9), 120.0 ± 40.9 min; near-total thyroidectomy with central lymph node dissection (n = 27), 159.9 ± 45.5 min; subtotal thyroidectomy (n = 29), 130.7 ± 50.2 min; subtotal thyroidectomy with central lymph node dissection (n = 2), 170.3 ± 35.4 min; lobectomy (n = 306), 104.6 ± 35.1 min; and lobectomy with central lymph node dissection (n = 25), 128.0 ± 35.2 min (Table 2).

**Table 2:** Extent of surgery and mean operation time (min)

**Extent of surgery**	**Number**	**Mean operation time (min)**
Total thyroidectomy	3 (0.7%)	156.6 ± 48.0
Total thyroidectomy with CLND*	47 (10.4%)	171.6 ± 38.9
Near-total thyroidectomy	9 (2.0%)	120.0 ± 40.9
Near-total thyroidectomy with CLND	27 (6.0%)	159.9 ± 45.5
Subtotal thyroidectomy	29 (6.4%)	130.7 ± 50.2
Subtotal thyroidectomy with CLND	2 (0.4%)	170.3 ± 35.4
Thyroid lobectomy	306 (67.4%)	104.6 ± 35.1
Thyroid lobectomy with CLND	25 (5.8%)	128.0 ± 35.2

*, central lymph node dissection.

The characteristics of the 128 patients who underwent endoscopic thyroidectomy are summarized in Table 3. The mean tumour size was 1.05 ± 0.79 cm. The tumours were located in the right lobe, left lobe, isthmus, and bilateral lobes in 43.8%, 40.6%, 5.5%, and 9.4% of the patients, respectively. Multifocal tumours were identified in 17.2% of patients. Extrathyroidal extensions and lymph node metastases were present in 6 (4.7%) and 76 (59.4%) patients, respectively. A total of 109 (92.2%) patients had stage I disease, and 9 (7.8%) patients had stage III disease. The mean follow-up period was 54.0 ± 35.7 months (range 6-160).

**Table 3:** Clinicopathologic characteristics of the patients with differentiated thyroid carcinoma (n = 128)

**Characteristics**		**Number**
Tumour size (cm)		1.05 ± 0.79
Tumour location	Right	56 (43.8%)
	Left	52 (40.6%)
	Isthmus	7 (5.5%)
	Bilateral	12 (9.4%)
Capsule invasion		26 (21.3%)
Extrathyroidal extension		6 (4.7%)
Lymphatic invasion		22 (20.0%)
Multiplicity		22 (17.2%)
Central lymph node metastasis		76 (59.4%)
Retrieved central lymph nodes		6.15 ± 4.90 (range 1-26)
Metastatic central lymph nodes		3.53 ± 2.78 (range 1-16)
TNM stage	I	106 (92.2%)
	II	0
	III	9 (7.8%)
Mean follow-up period (months)		54.0 ± 35.7 (range 6-160)

Temporary and permanent hypoparathyroidism that required calcium and vitamin D supplementation occurred in 32 (7.1%) and 4 (0.9%) of the 452 patients, respectively. Transient vocal cord paresis occurred in 20 (4.4%) patients. Postoperative uncontrolled bleeding occurred in 2 cases and was successfully controlled by endoscopic surgery. No complications, such as tracheal injury, oesophageal perforation, or flap perforation, occurred during the operations (Table 4). 1 case were converted to a conventional open thyroidectomy because of uncontrolled bleeding.

**Table 4:** Postoperative complications (n = 452)

**Complications**		**Number**
Hypoparathyroidism	Temporary	32 (7.1%)
	Permanent	4 (0.9%)
Recurrent laryngeal nerve injury	Temporary	20 (4.4%)
	Permanent	0
Bleeding		2 (0.4%)
Tracheal injury		0
Oesophageal injury		0
Wound infection		3 (0.7%)

In 128 patients with differentiated thyroid carcinoma, 72 patients underwent total thyroidectomy or near-total thyroidectomy. Postoperative thyroglobulin(Tg) levels were available for 60 of the 72 patients. Mean postoperative Tg level was 0.78 ng/ml. 47 of these patients (78.3%) had T4-suppressed level of Tg < 1.0 ng/ml.

## Discussion

In accordance with the increased utilization of ultrasonography, the worldwide incidence of thyroid nodules and carcinoma has progressively increased in recent decades [5]. Because the incidence of thyroid carcinoma in young women is rapidly increasing, cosmesis plays an important role in thyroid operations. In addition to improving the cosmetic results, endoscopic thyroidectomy can reduce the postoperative hospital stay and postoperative pain. Thus, various endoscopic thyroid surgical approaches have been performed, including cervical [6], anterior chest wall [7], axilla [8,9], axilla-breast [10,11], and breast [12,13]. While endoscopic thyroid surgery has advantages over other techniques, endoscopic thyroid surgery presents disadvantages for surgeons with limited experience. These disadvantages include a longer learning curve, longer operation time, and the potential for more severe injuries compared to the conventional procedure; however, these disadvantages can be overcome by experienced surgeons.

We have performed endoscopic thyroid surgeries via a breast approach since 1999. Compared with various endoscopic thyroid surgery techniques, such as conventional open thyroidectomy, the breast approach has the advantage of presenting a good visual field. Because a 5-mm endoscope is inserted into the right parasternal region, both thyroid lobes, the recurrent laryngeal nerves, the upper and lower parathyroid glands, and the thyroid vessels can be identified with a symmetrical view. With respect to cosmesis, patient satisfaction is high. The circumareolar wounds of the breast nipples and the 3-mm working port wound heal extremely well and are rarely visible after the procedure unless hypertrophic scars or keloids form.

When contraindications are absent, endoscopic thyroidectomy has recently been applied for benign thyroid tumours. However, safety guidelines for endoscopic thyroidectomy for thyroid cancer have not been published. Thyroid cancer was initially considered to be a contraindication for endoscopic thyroidectomy [14]. However, because differentiated thyroid cancers exhibit good prognoses, endoscopic thyroidectomies for low-risk thyroid cancer are increasingly being performed. With the advent of new surgical skills and the accumulation of a surgeon’s experiences, the indications for endoscopic thyroidectomy are being extended. Currently, the indications for endoscopic thyroidectomy are as follows: patients younger than 45 years old; well-differentiated thyroid cancer of less than 2 cm in diameter; no definite evidence of local invasion or central lymph node metastasis; no adhesion or fixation of enlarged lymph nodes around the neck compartment; and patients who are interested in endoscopic thyroid surgery [15]. We also performed endoscopic thyroidectomy for malignant thyroid carcinoma with the above-mentioned indications.

In 90 out of the 128 patients who underwent endoscopic thyroidectomy for thyroid carcinoma, ipsilateral or bilateral central lymph node dissections were performed with no risk of complications. In our study, the mean number of retrieved lymph nodes was 6.15 ± 4.90 in the central compartment. In patients with thyroid carcinoma, central lymph node dissection is feasible using our surgical technique.

The rates of transient and permanent hypoparathyroidism after conventional open thyroidectomy range from 0.3% to 49% and 0% to 14.3%, respectively, and the rate of unintentional recurrent laryngeal nerve injury ranges from 0% to 5.7% [16-18].

The results of this study were similar. Transient and permanent hypoparathyroidism were observed in 7.1% and 0.9% of patients, respectively. Transient and permanent recurrent laryngeal nerve injuries were observed in 4.4% and 0% of patients, respectively. Compared with conventional open thyroidectomy, our surgical technique does not differ with respect to the incidences of hypoparathyroidism and recurrent laryngeal nerve injury.

There were no life-threatening operative complications after the endoscopic thyroidectomies. While there were cases of immediate postoperative bleeding (2 cases) and wound infection (3 cases), these complications were corrected without difficulty. Tracheal injury, oesophageal injury, flap injury, and subcutaneous emphysema were not observed.

In this study, 2 out of 128 patients developed recurrent thyroid carcinoma after endoscopic thyroidectomy. One patient underwent endoscopic total thyroidectomy with central lymph node dissection. Tumour histology revealed papillary carcinoma. The tumour size was 2.2 cm, and extrathyroidal extension was not observed, but central lymph node metastasis was observed. The patient was treated with radioactive iodine therapy. After 28 months, the follow-up imaging study revealed metastases to the central and right lateral lymph nodes. FNAC was performed in the right lateral lymph node, which supported the diagnosis of papillary carcinoma. Therefore, we performed a modified radical neck dissection. Another patient underwent endoscopic lobectomy in September 2000. Tumour histology revealed papillary microcarcinoma. The tumour was 0.5 cm in size. The patient was treated with levothyroxine for serum thyrotropin (TSH) suppression. After 132 months, a newly developed lesion was observed in the contralateral thyroid lobe. We performed open completion thyroidectomy with central lymph node dissection. To date, no mortality has been reported in these patients. Thus, endoscopic thyroidectomy for thyroid carcinoma is safe, especially in cases of low-risk differentiated thyroid carcinoma.

## Conclusions

Endoscopic thyroidectomy via a breast approach is a safe, feasible, and minimally invasive surgical method for benign and low-risk malignant thyroid disease. This approach provides good operative results and has a low complication rate. For selected thyroid disease patients who worry about neck scars, endoscopic thyroidectomy via a breast approach is an effective surgical option.

## Abbreviations

FNAC: Fine needle aspiration cytology; TNM: Tumour-node-metastasis; AJCC: American Joint Committee on Cancer.

## Competing interests

The authors declare that they have no competing interests

## Authors’ contributions

YSK: Study conception and design; Analysis and interpretation of data; Drafting of manuscript. KHJ: Analysis and interpretation of data. SCP: Analysis and interpretation of data. KHK: Analysis and interpretation of data. CHA: Analysis and interpretation of data. JSK: Study conception and design; Critical revision. All authors read and approved the final manuscript

## Pre-publication history

The pre-publication history for this paper can be accessed here:

http://www.biomedcentral.com/1471-2482/14/49/prepub
